# Association Between the Number of Metabolic Syndrome Components and Markers of Subclinical Vascular Damage: A Retrospective Cross-Sectional Study

**DOI:** 10.3390/jcm15176638

**Published:** 2026-08-28

**Authors:** Monika Starzak, Grzegorz K. Jakubiak, Natalia Pawlas, Grzegorz Cieślar, Agata Stanek

**Affiliations:** 1 Department of Internal Medicine, Angiology and Physical Medicine, Faculty of Medical Sciences in Zabrze, Medical University of Silesia in Katowice, Batorego 15 St., 41-902 Bytom, Poland; cieslar1@tlen.pl; 2 Department of Pharmacology, Faculty of Medical Sciences in Zabrze, Medical University of Silesia in Katowice, Jordana 38 St., 41-808 Zabrze, Poland; grzegorz.k.jakubiak@gmail.com (G.K.J.); n-pawlas@wp.pl (N.P.); 3 Department of Internal Medicine, Metabolic Diseases and Angiology, Faculty of Health Sciences in Katowice, Medical University of Silesia in Katowice, Ziolowa 45/47 St., 40-635 Katowice, Poland; astanek@tlen.pl

**Keywords:** metabolic syndrome, atherosclerosis, arterial stiffness, pulse wave velocity, pulse wave analysis, ankle–brachial index, toe–brachial index, intima–media thickness

## Abstract

**Background:** Metabolic syndrome (MetS) refers to the coexistence of cardiometabolic disorders, such as hyperglycemia, elevated blood pressure, visceral obesity, or features of atherogenic dyslipidemia. A diagnosis of MetS significantly increases the risk of cardiovascular disease (CVD). However, the exact relationship between the number of MetS components, regardless of their constellation, and features of subclinical cardiovascular dysfunction remains unclear. This study aimed to explore the relationship between the number of MetS components and features of subclinical atherosclerosis and arterial stiffness (AS) in patients without clinically overt CVD. **Methods:** This retrospective study included 100 patients (mean age: 58.8 ± 16.81 years), without signs of acute illness or diagnosed atherosclerotic CVD, who were admitted to a clinic for scheduled internal medicine diagnostics. Participants were assessed for MetS components and underwent cardiovascular tests, including Doppler ultrasonography of the carotid, vertebral, and lower limb arteries; measurement of intima–media thickness (IMT) in the carotid (cIMT), common femoral (cfIMT), and superficial femoral (sfIMT) arteries; and assessment of AS using carotid–femoral pulse wave velocity (cfPWV) and pulse wave analysis (PWA). **Results:** Univariate analysis revealed significant correlations between the number of MetS components and IMT in all vascular beds: cIMT (R = 0.371; *p* < 0.001), cfIMT (R = 0.343; *p* < 0.001), and sfIMT (R = 0.516; *p* < 0.001). Significant correlations were also found for several AS and PWA parameters: cfPWV (R = 0.398; *p* < 0.001), central pulse pressure (cPP) (R = 0.223; *p* < 0.026), and end-systolic pressure (ESP) (R = 0.241; *p* = 0.016). Meanwhile, in the multivariable analysis adjusted for sex, age, and body mass index (BMI), only the association between the subendocardial viability ratio (SEVR) and the number of MetS components was significant (β = 0.33; 95% CI 0.084–0.577; *p* = 0.009); however, this association does not remain significant after Benjamini–Hochberg correction (*p* = 0.108). **Conclusions:** Although the number of MetS components correlated significantly with several parameters of subclinical atherosclerosis and AS, most associations were explained by confounding variables. These results do not support the use of a simple count of MetS components as an indicator of subclinical cardiovascular dysfunction related to atherosclerosis and AS.

## 1. Introduction

Arterial stiffness (AS) represents an early stage in the development of cardiovascular disease (CVD). The loss of vascular elasticity impairs arterial hemodynamic function and increases cardiac workload, contributing to hypertension, ischemic heart disease, and heart failure progression in its initial stages [1,2]. Estimation of individual CVD risk is strongly recommended. However, assessing conventional risk factors and biomarkers such as smoking status, age, blood pressure, and total cholesterol level may have limited effectiveness. An additional assessment of AS could improve risk stratification [3].

Several studies have shown that obesity is a significant factor in AS onset [4,5,6]. Body mass index (BMI), fat mass, and visceral fat thickness have been identified as the variables most strongly associated with AS [7]. Moreover, dyslipidemia, including elevated triglyceride levels, as well as impaired glucose metabolism with insulin resistance and elevated fasting serum glucose concentrations, contribute to endothelial and microvascular damage and, subsequently, to features of AS [8,9]. Metabolic syndrome (MetS), defined as a cluster of coexisting metabolic dysfunctions, with obesity as the primary component, contributes to the progression of AS and atherosclerosis [10]. The SPARTE study showed that hypertension treatment based on AS resulted in better outcomes than therapy based on blood pressure control alone [11].

Although the individual components of MetS have been extensively studied in terms of their contribution to atherosclerosis and AS development, the relationship between the simple number of MetS components and the presence and severity of subclinical cardiovascular dysfunction remains unclear. A better understanding of this relationship, particularly in patients without clinically overt atherosclerotic CVD, could improve CVD risk assessment and guide targeted preventive strategies in clinical practice. We recently reported the results of a retrospective analysis assessing the relationship between the number of MetS components and features of subclinical cardiac dysfunction, as evaluated using transthoracic echocardiography, in individuals without previously diagnosed CVD [12]. Using data collected from the same patient population, the present analysis focuses on features of subclinical atherosclerosis and AS.

The purpose of this study was to evaluate the correlation between the number of MetS components and selected parameters of subclinical atherosclerosis and AS in patients without clinically overt atherosclerotic CVD. Unlike studies comparing participants solely according to the presence or absence of MetS, this exploratory analysis assessed the cross-sectional association between the number of MetS components and multiple vascular measurements obtained from different vascular beds. The study did not assess the cumulative biological contribution of individual components, temporal progression, or causality.

## 2. Materials and Methods

### 2.1. Study Population

This study was a retrospective analysis of data collected from patients who underwent scheduled internal medicine diagnostics at the Department of Internal Medicine, Angiology and Physical Medicine, Medical University of Silesia, between June 2022 and February 2024.

Each participant was subjected to at least three of the following procedures: (1) measurement of the ankle–brachial index (ABI) and toe–brachial index (TBI); (2) measurement of central blood pressure using pulse wave analysis (PWA) and of carotid–femoral pulse wave velocity (cfPWV); (3) Doppler ultrasonography of the carotid and vertebral arteries; and (4) Doppler ultrasonography of the lower limb arteries.

Patients were included consecutively if measurements from at least three of the aforementioned procedures were available. Patients were excluded if they were missing vascular measurements, had incomplete clinical data, or met the predefined exclusion criteria [12] presented in Table 1.

### 2.2. Anthropometric and Laboratory Measurements

Basic anthropometric measurements included body mass (M), body height (H), waist circumference (WC), and hip circumference (HC). Based on these measurements, BMI, waist-to-hip ratio (WHR), and waist-to-height ratio (WHtR) were calculated according to the following formulas:

(1) $$BMI[kg/m^{2}]=\frac{M\left[kg\right]}{{\left(H\left[m\right]\right)}^{2}}\;\;\;\;$$

(2) $$WHR=\frac{WC[cm]}{HC[cm]}\;\;\;\;$$

(3) $$WHtR=\frac{WC[cm]}{H[cm]}\;\;\;$$

Basic anthropometric measurements were supplemented with body composition analysis using a TANITA MC-780 device (Tanita Europe B.V., Amsterdam, The Netherlands).

Blood samples were collected between 6:00 and 8:00 a.m. after a fasting period of at least 14 h. All laboratory tests were conducted at the Laboratory of Specialist Hospital No. 2 in Bytom.

### 2.3. Definition of Metabolic Syndrome

MetS components were diagnosed in accordance with the definition published in 2009 as a consensus statement of the International Diabetes Federation Task Force on Epidemiology and Prevention; National Heart, Lung, and Blood Institute; American Heart Association; World Heart Federation; International Atherosclerosis Society; and International Association for the Study of Obesity [13]. The criteria for diagnosing individual MetS components are presented in Table 2.

### 2.4. Cardiovascular Assessment

The following assessments were performed to evaluate the cardiovascular system: Doppler ultrasonography of the carotid and vertebral arteries and the lower extremity arteries with intima–media thickness (IMT) measurements in the carotid (cIMT), common femoral (cfIMT), and superficial femoral (sfIMT) arteries; cfPWV measurement; PWA; and ABI and TBI measurements. The following PWA parameters were included in the final analysis: central pulse pressure (cPP), augmentation pressure (AP), augmentation index (AIx), augmentation index normalized to a heart rate of 75 beats per minute (AIx75), heart rate duration (HRD), ejection duration (ED), subendocardial viability ratio (SEVR), and end-systolic pressure (ESP).

Doppler ultrasonography was performed according to the current guidelines and recommendations [14,15,16]. IMT was measured manually, and for each anatomical location, measurements were performed three times. The arithmetic mean was recorded in the patient’s medical history for each site. The mean of the measurements obtained on both sides was analyzed as cIMT, cfIMT, or sfIMT, as appropriate. cIMT was measured at the posterior wall of the common carotid artery, 1–2 cm below the bifurcation, while cfIMT and sfIMT were measured at the posterior wall of the common femoral artery and superficial femoral artery, 1–2 cm above or below the bifurcation, respectively. IMT was measured as described regardless of the presence of atherosclerotic plaque at these sites. All IMT measurements were performed by the same physician, who was experienced in performing Doppler ultrasonography.

Atherosclerotic plaque was defined as a focal structure encroaching into the arterial lumen by at least 0.5 mm or 50% of the surrounding IMT value, or demonstrating a thickness > 1.5 mm, measured from the media–adventitia interface to the intima–lumen interface [17]. Information on plaque morphology and exact plaque dimensions was not systematically recorded and was therefore unavailable for the present retrospective analysis.

All tests were performed with participants in the supine position, under thermally comfortable conditions, after at least 10 min of rest.

The equipment used for cardiovascular assessment is presented in Table 3.

### 2.5. Statistical Analysis

Qualitative variables were presented as absolute numbers and percentages, while quantitative variables were assessed for normality using the Shapiro–Wilk test and by visually inspecting histograms. Variables with an approximately normal distribution were presented as mean ± standard deviation (SD), whereas variables with non-normal distributions were presented as median and interquartile range (Q1; Q3).

Between-group comparisons were performed using Student’s *t*-test for normally distributed continuous variables and the Mann–Whitney U test for non-normally distributed continuous variables. For qualitative variables, the χ^2^ test was used. Correlations between the number of MetS components and cardiovascular parameters were assessed using Spearman’s rank correlation coefficient.

Multivariable associations between the number of MetS components and individual vascular parameters were evaluated using multiple linear regression models. Age, sex, and BMI were included a priori as potential confounding variables because of their established associations with vascular structure and function. Variables with markedly non-normal distributions were logarithmically transformed before inclusion in the regression models; these included cfIMT, sfIMT, cfPWV, cPP, and AP. ABI could not be adequately normalized and was therefore not included in the multivariable regression analysis.

For each regression model (generalized linear models), the standardized regression coefficient (β) for the number of MetS components, its 95% confidence interval (95% CI), and the corresponding *p*-value were reported. Overall model performance was assessed using the coefficient of determination (R^2^) and the omnibus model *p*-value.

Because multiple vascular outcomes were evaluated in the primary multivariable analysis, the Benjamini–Hochberg procedure was applied to control the false discovery rate across the twelve vascular outcomes included in this analysis. Nominal statistical significance was defined as *p* < 0.05, whereas statistical significance after correction for multiple testing was defined as a Benjamini–Hochberg-adjusted *p* < 0.05.

Because the association between the number of MetS components and SEVR observed in the fully adjusted model was unexpected, additional regression models incorporating different combinations of covariates were performed as exploratory sensitivity analyses. These models included the unadjusted model and models adjusted for sex; age; BMI; age and BMI; and sex, age, and BMI. These additional analyses were considered exploratory and were performed to assess the robustness and direction of the observed association rather than to provide confirmatory evidence.

As a consequence of the retrospective study design, the number of available observations differed slightly between individual vascular parameters. Missing data resulted primarily from incomplete documentation or technical limitations affecting the quality or interpretability of selected measurements. Analyses were performed using an available-case approach; therefore, each correlation analysis and multivariable model included participants with complete data for all variables required for that specific analysis. No imputation of missing data was performed. The number of observations included in each analysis is reported in the corresponding tables.

Statistical analyses were performed using Statistica version 13 (TIBCO Software Inc., Palo Alto, CA, USA).

### 2.6. Ethical Information

The present study was based on a retrospective data analysis and therefore did not require approval from a bioethics committee. A written request for confirmation of this fact was submitted to the Bioethics Committee of the Medical University of Silesia in Katowice, which received an affirmative response (6 February 2024, BNW/NWN/0052/KB/19/24).

## 3. Results

### 3.1. Characteristics of the Study Population

A total of 100 participants, including 63 women and 37 men, were included in the final analysis. Their mean age was 58.8 ± 16.81 years, the mean BMI was 27.01 ± 5.02 kg/m^2^, the mean WHR was 0.91 ± 0.095, and the mean WHtR was 0.56 ± 0.092. Overall, 35 participants (35.0%) were overweight and 29 (29.0%) had obesity. The mean body fat mass was 23.03 ± 8.62 kg, and the mean body fat percentage was 29.86 ± 7.55%.

Hypertension was the most common comorbidity, affecting 63 participants (63.0%). Disorders of carbohydrate metabolism were present in 43 participants (43.0%), including 22 (22.0%) with diabetes and 21 (21.0%) with prediabetes. Chronic kidney disease and atrial fibrillation were present in 2.0% and 5.0% of participants, respectively. Current smokers accounted for 43.4% of the study population, former smokers accounted for 27.3%, and never-smokers accounted for 29.3%; smoking-status data were unavailable for one participant. The median triglyceride, non-HDL cholesterol, uric acid, and glycated hemoglobin levels were 118.5 mg/dL, 131.85 mg/dL, 5.0 mg/dL, and 5.61%, respectively.

Forty-five participants (45.0%) fulfilled the diagnostic criteria for MetS, and the number of MetS components ranged from 0 to 5. A total of 12 participants (12.0%) had no MetS components, 15 (15.0%) had one component, 28 (28.0%) had two components, 22 (22.0%) had three components, 13 (13.0%) had four components, and 10 (10.0%) had all five components.

Upon comparing participants with and without MetS, those with MetS were significantly older, had higher BMI, WC, and WHR, and had greater body fat mass and a higher body fat percentage. Detailed characteristics of the groups with and without MetS are presented in Table 4.

The characteristics of this cohort have also been reported in a previous publication based on the same study population [12].

### 3.2. Cardiovascular Parameters

Atherosclerotic plaque was detected in 66% of patients, but no participant had hemodynamically significant stenosis. Because exact plaque location, dimensions, and morphology were not systematically recorded in the analytical database, these characteristics could not be evaluated retrospectively. An ABI < 1.0 was found in 17% of patients, and an ABI < 0.9 in 8%. No participants had an ABI > 1.3. A cfPWV > 10 m/s was found in 24.74% of patients. Complete descriptive statistics for the parameters of subclinical atherosclerosis and AS are presented in Table 5.

### 3.3. Correlation Between Cardiovascular Parameters and Number of Metabolic Syndrome Components

IMT in all vascular beds was significantly linked to the number of MetS components. However, no significant correlation was found for ABI or TBI. cfPWV and cPP were found to be significantly correlated with the number of MetS components. Among the central hemodynamic and PWA-derived parameters, significant correlations were observed for cPP and ESP. The complete results of the correlation analysis between cardiovascular parameters and the number of MetS components are presented in Table 6.

### 3.4. Comparison of Patients with and Without Detectable Atherosclerotic Plaque

Patients with detectable atherosclerotic plaque had a significantly higher median number of MetS components. Participants with detectable plaque also had significantly higher median IMT values in all vascular beds, as well as higher cfPWV, cPP, and AP values. Median ABI was significantly lower in patients without atherosclerotic plaque. Mean AIx, AIx75, and ED values were significantly higher in patients with atherosclerosis. No significant differences were found for TBI, HRD, SEVR, or ESP. The complete results of the comparison between patients with and without atherosclerosis are presented in Table 7.

### 3.5. Multivariate Analysis

The constructed multivariate analysis model, adjusted for age, sex, and BMI, was significant for cIMT, TBI, AIx, AIx75, HRD, ED, SEVR, and ESP, as well as for log(cfIMT), log(sfIMT), log(cfPWV), log(cPP), and log(AP). The ABI variable could not be normalized. The complete results regarding the significance of the models explaining the individual variables are presented in Table 8.

Among the parameters assessed, the number of MetS components contributed significantly to explaining the variability in SEVR, but not in the other parameters, in the model adjusted for age, sex, and BMI. However, the result for the relationship between the number of MetS components and SEVR was unexpected, because it indicated that SEVR increased with the number of MetS components (β = 0.33; 95% CI: 0.084–0.577; *p* = 0.009). Benjamini–Hochberg correction was applied across the 12 vascular outcomes included in Table 8. Nominal statistical significance was defined as *p* < 0.05 and statistical significance after correction as q < 0.05. The nominal association with SEVR did not remain statistically significant after correction. Table 9 presents detailed results regarding the contribution of the number of MetS components to explaining the variability in individual parameters of subclinical atherosclerosis and AS.

### 3.6. Further Analysis of the Relationship Between the Number of Metabolic Syndrome Components and the Subendocardial Viability Ratio

**Figure 1:**
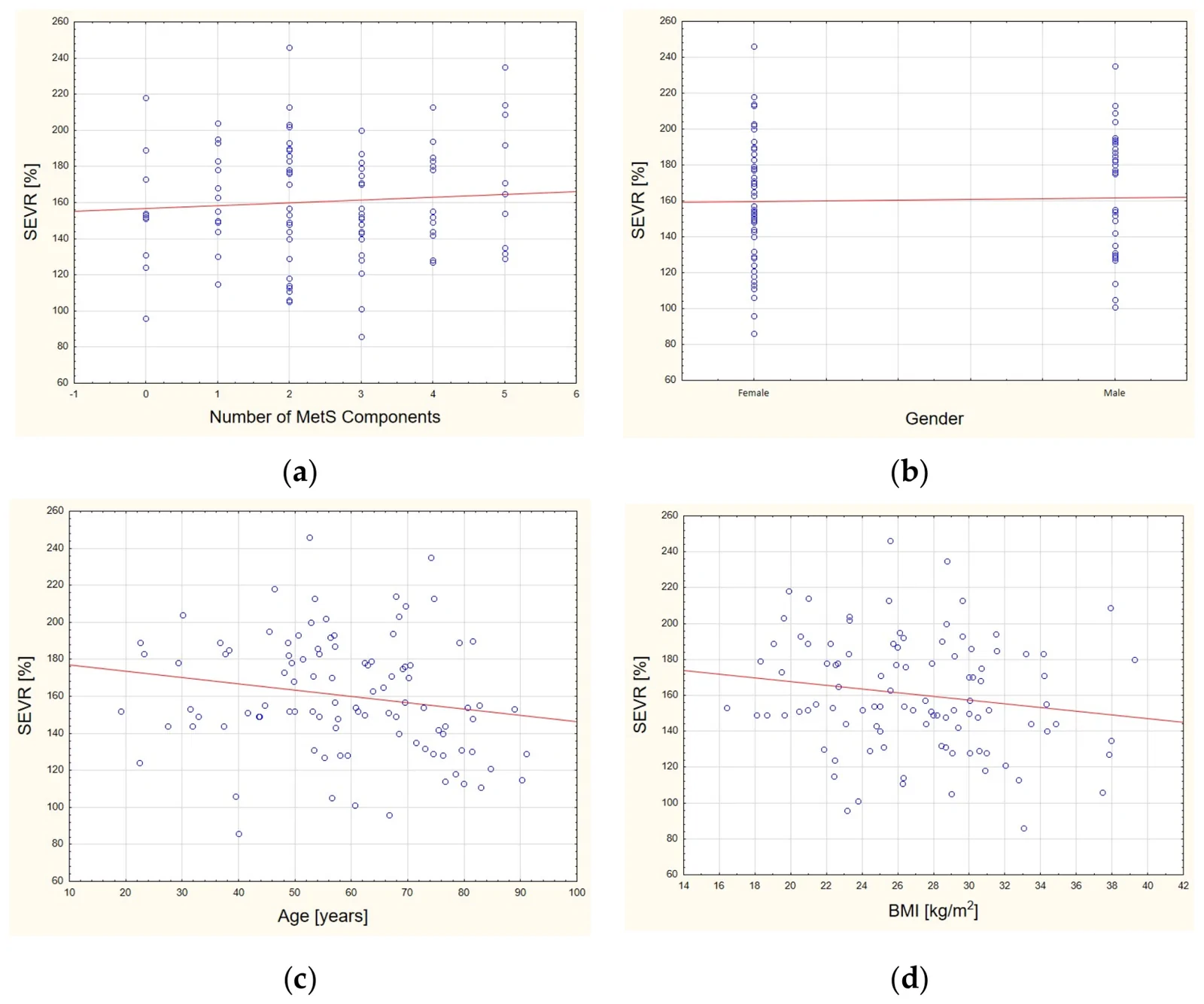
Relationship between the subendocardial viability ratio (SEVR) and (**a**) the number of metabolic syndrome (MetS) components, (**b**) sex, (**c**) age, and (**d**) body mass index (BMI). Blue circles represent individual observations, and red lines indicate linear regression lines.

Correlation analysis revealed a significant correlation between SEVR and age (R = −0.234; *p* = 0.019), but not between SEVR and BMI (R = −0.175; *p* = 0.081). As previously reported, no significant correlation was found between SEVR and the number of MetS components, although multivariable analysis adjusted for age, sex, and BMI revealed a significant positive association between these variables. SEVR values did not differ significantly between men and women (161.68 ± 32.66% vs. 159.52 ± 31.1%; *p* = 0.744). The number of MetS components was significantly correlated with age (R = 0.362; *p* < 0.001) and BMI (R = 0.57; *p* < 0.001). Figure 1 graphically presents the correlations between SEVR and the number of MetS components, sex, age, and BMI.

To better understand the preceding results regarding the relationship between SEVR and the number of MetS components, additional models incorporating different combinations of confounding variables were constructed. No significant model was obtained in the unadjusted analysis or after adjustment for sex or age alone. The model adjusted for BMI was statistically significant, but the number of MetS components did not contribute significantly to explaining variability in SEVR. Only in the model adjusted for age and BMI did the number of MetS components contribute significantly to explaining SEVR variability; the results were similar to those obtained in the model adjusted for age, sex, and BMI. The complete results are presented in Table 10.

## 4. Discussion

Cardiovascular risk assessment is essential for reducing CVD morbidity and mortality in patients with MetS; therefore, identifying subclinical atherosclerosis and AS is crucial for establishing preventive strategies. Increased AS and coronary calcification in patients without overt CVD or diabetes are associated with high cardiovascular risk according to SCORE2 [18,19]. Although a greater number of MetS components was associated with several markers of vascular injury in the unadjusted analyses, these associations were substantially attenuated after adjustment for age, sex, and BMI. This finding suggests that the crude relationship between the cumulative burden of MetS components and vascular abnormalities is largely influenced by these major demographic and anthropometric determinants. Therefore, a simple count of MetS components may not represent an independent predictor of vascular damage, but may instead reflect the effects of aging and excess adiposity. Both aging and obesity are well-established contributors to arterial remodeling, endothelial dysfunction, and increased AS. Obesity promotes chronic low-grade inflammation, oxidative stress, insulin resistance, and vascular dysfunction, whereas aging is associated with progressive structural and functional changes in the arterial wall. Consequently, age and obesity may account for a substantial proportion of the vascular alterations attributed to an increasing number of MetS components. These findings also indicate that the cumulative number of MetS components should be interpreted as a pragmatic measure of overall metabolic burden rather than a surrogate for the independent effects of individual metabolic abnormalities.

cIMT is recognized as a marker of subclinical atherosclerosis due to its established association with myocardial infarction and ischemic stroke, irrespective of classic cardiovascular risk factors [20]. Moreover, in the ARIC study, higher systolic and diastolic blood pressure values correlated with increased cIMT regardless of sex [21]. The coherence between cIMT and diabetes is well established. Additionally, in non-diabetic patients, cIMT has also been linked with glucose intolerance, impaired fasting glucose, and impaired glucose tolerance two hours after a glucose load. However, the correlation between cIMT and fasting glucose remains inconclusive [22]. Patients with diabetes who received intensive multifactorial intervention and achieved optimal blood glucose, blood pressure, and lipid levels exhibited a significant reduction in atherosclerotic plaque development and an overall regression in mean cIMT [23]. The link between cIMT and obesity was particularly evident in women and was primarily related to WC and BMI [24]. However, cIMT may also increase with age, without atherosclerosis progression [25]. Thus, there is disagreement among researchers regarding the association between conventional CV risk factors and cIMT [26].

Several methods and clinical indices are used to assess AS in clinical practice. cfPWV, measured using standard methods such as tonometric carotid–femoral measurement, remains the gold-standard marker of AS [27]. The Framingham study demonstrated that cfPWV increases CVD morbidity when incorporated with a base model which included age and sex [28]. In addition, both conventional risk markers and cfPWV have proven valuable for predicting CVD, and they may support preventive interventions in healthy populations and in patients with cardiovascular comorbidities or MetS components. Vascular age, when estimated from brachial–ankle cfPWV, has been proven to be substantially higher than participants’ actual chronological age [29], and cfPWV has been found to be significantly correlated not only with the presence of coronary artery calcification and coronary artery atherosclerosis, but also with their severity [30]. However additional parameters should be taken into consideration when assessing AS in patients with MetS components. The augmentation index (AIx), derived from the arterial pressure waveform, represents the contribution of the reflected pressure wave to central systolic pressure. AIx is influenced by age, sex, heart rate, blood pressure, and height [31,32], and is affected by macrovascular properties and microvascular function, as described by systemic vascular resistance. Several studies have shown that AIx is a composite hemodynamic marker, rather than a specific marker of AS alone. In patients with MetS components, AIx should therefore be taken into account and interpreted in the context of AS rather than as a stand-alone marker [33,34]. Arterial elastic properties, pressure wave reflection from peripheral vessels, and wave propagation along the arteries determine pulse pressure amplification (PPA). Reduced PPA provides complementary information alongside cfPWV regarding increased central hemodynamic stress, premature vascular aging, and AS. Protogerou et al. showed that patients with MetS had higher PPA, HRD, and cfPWV values than age-, sex-, and blood-pressure-matched patients without MetS. However, this distinction was no longer observed after adjustment for heart rate and cfPWV [35]. The authors reported PPA to be related to increased risk for coronary artery disease but not for cerebrovascular disease.

The subendocardial viability ratio (SEVR), also referred to as the Buckberg index, assessed via applanation tonometry, estimates the balance between myocardial oxygen supply and demand. It is a noninvasive marker of coronary perfusion, myocardial workload, oxygen supply, and subclinical atherosclerosis, and is calculated as the ratio of the diastolic pressure time index (DPTI) to the systolic pressure time index (SPTI, also known as the tension–time index (TTI) or Sarnoff index). Reduced SEVR values indicate impaired subendocardial perfusion [36,37], and several studies have shown that SEVR is reduced in patients with hypertension or peripheral artery disease [38,39]. Moreover, SEVR has been identified as a predictor of cardiovascular complications in individuals with chronic kidney disease [40]. Previous research has shown reduced SEVR in patients with MetS, as well as in individuals who do not meet the criteria for MetS but have isolated metabolic abnormalities such as hyperglycemia, obesity, or dyslipidemia [41]. Early vascular impairment is a frequent feature of MetS, and lower SEVR values together with increased IMT have already been documented [42]. Diabetic patients also exhibit increased values of cfPWV and, thus, greater AS, and reduced SEVR values have been observed in patients with diabetes or impaired fasting glucose compared with normoglycemic patients [43]. Reduced SEVR in patients with MetS may represent an important factor in increased CVD risk. Higher SEVR values have been associated with a significantly lower risk of major adverse cardiovascular events (MACEs), with the strongest association observed in older individuals [44]. Our study did not confirm a significant unadjusted association between the number of MetS components and SEVR. General linear models revealed an unexpected positive association between these variables, but only in models adjusted for age and BMI or for age, sex, and BMI. This suggests that the observed association may reflect the specific characteristics of the study population rather than a general relationship. Further studies are required to better understand the association between the number of MetS components and SEVR.

Our findings suggest that the number of MetS components is associated with selected vascular parameters in unadjusted analyses; however, these associations did not remain significant in multivariable analyses. Thus, the results do not support the number of MetS components as a simple marker of subclinical cardiovascular dysfunction. These findings indicate that the relationship between the burden of MetS and subclinical vascular damage is complex and strongly influenced by major clinical confounders. The observed association with SEVR may represent a relevant marker linking metabolic abnormalities and the imbalance between myocardial oxygen supply and demand, and warrants further investigation in larger prospective studies.

Furthermore, the simple count of MetS components may not fully capture vascular risk, as it assigns equal weight to conditions that differ markedly in severity and duration, such as mildly elevated triglycerides and long-standing diabetes or hypertension; therefore, analyses considering the specific composition and clinical burden of individual MetS components may provide more informative insights.

### Limitations of This Study

This research has several limitations. First, it was a retrospective single-center study, with the study population consisting of hospitalized patients referred for elective diagnostic evaluation, which may have introduced selection bias and limited the generalizability of the findings. The absence of a healthy control group precluded direct comparisons between individuals with and without metabolic abnormalities, and the analysis was based on data collected at a single time point, making it impossible to assess temporal relationships between the investigated variables. Furthermore, the cross-sectional design allowed the identification of certain relationships, but not of cause-and-effect relationships. Information on the duration of individual metabolic disorders and on the effects of ongoing pharmacological treatment was not incorporated into the analyses. Consequently, residual confounding related to disease chronicity and treatment-related modification of vascular parameters cannot be excluded. The cumulative number of MetS components was used as a measure of metabolic burden. Although this approach is clinically simple and widely used, it assumes that all components contribute equally to cardiovascular risk. In reality, individual MetS components differ in their biological effects, severity, duration, and treatment status, and these factors may affect vascular damage to different degrees. Therefore, the number of MetS components may not fully capture the complexity of metabolic risk. Future studies involving larger populations should evaluate the independent and combined effects of individual MetS components, as well as their severity and duration, on vascular remodeling. The study database recorded the presence of atherosclerotic plaque and the degree of associated stenosis, but did not contain systematic information on plaque’s exact location, dimensions, number, or morphology. Consequently, plaque burden and territory-specific associations could not be assessed. In addition, IMT was measured regardless of the presence of plaque at the measurement site, which may explain the high maximum femoral-wall-thickness values and limit their interpretation as conventional plaque-free IMT. The relatively small sample size may also have reduced statistical power and prevented more detailed subgroup analyses. Moreover, due to the retrospective nature of this analysis, there were some gaps in the dataset, and while these gaps were relatively minor, the incomplete availability of selected vascular parameters may have reduced the statistical power of some analyses; therefore, a potential influence of missing data on the results cannot be fully excluded.

Further studies should include larger and more diverse populations, preferably using a prospective design, with a more comprehensive evaluation of metabolic status, that incorporates additional markers of insulin resistance, inflammation, and detailed lipid profiles. Repeating similar analyses using alternative diagnostic criteria for MetS could also help determine the consistency and generalizability of the findings across different clinical definitions.

Finally, a prospective study with follow-up would enable an assessment of the identified associations’ prognostic significance and their impact on cardiovascular outcomes. The unexpected positive association between the number of MetS components and SEVR was sensitive to model specification and did not remain statistically significant after correction for multiple comparisons. It should therefore be interpreted as an exploratory, hypothesis-generating observation rather than as evidence of an independent relationship.

## 5. Conclusions

In this exploratory cross-sectional analysis, the number of MetS components was associated with several vascular parameters in the unadjusted analyses (IMT in various vascular beds, cfPWV, and cPP), but these relationships were largely attenuated after adjustment for age, sex, and BMI. The positive association with SEVR was nominally significant, but did not remain significant after correction for multiple comparisons and was sensitive to model specification. Therefore, the present findings do not support the use of a simple count of MetS components as an independent marker of subclinical vascular damage. Larger prospective studies are required to evaluate the effects of individual MetS components and their combinations on vascular structure and function.

## Figures and Tables

**Table 1:** Exclusion criteria [12].

Exclusion Criteria
Acute illness within the month preceding hospital admission
Diagnosed overt atherosclerotic cardiovascular disease in the medical history (myocardial infarction, stroke, or symptomatic peripheral artery disease)
Previous cardiovascular revascularization procedures in any vascular bed
Past implantation of a cardiac implantable device
Clinical symptoms of heart failure
Infection
Diagnosed autoimmune disease during immunosuppressive treatment
Diagnosed malignant neoplasm
Anemia

**Table 2:** Diagnostic criteria for particular components of metabolic syndrome (MetS) [13].

Component	Diagnostic Criteria
Abdominal obesity	Waist circumference ≥ 94 cm in men or ≥80 cm in women
Elevated triglycerides	Serum triglyceride concentration ≥ 150 mg/dL or pharmacological treatment of previously diagnosed hypertriglyceridemia
Decreased high-density lipoprotein cholesterol	Serum high-density lipoprotein cholesterol concentration < 40 mg/dL in men or <50 mg/dL in women, or pharmacological treatment of a previously diagnosed lipid disorder
Hyperglycemia	Fasting serum glucose concentration ≥ 100 mg/dL or pharmacological treatment of previously diagnosed diabetes or prediabetes
Elevated blood pressure	Systolic blood pressure ≥ 130 mmHg or diastolic blood pressure ≥ 85 mmHg, or pharmacological treatment of previously diagnosed arterial hypertension

**Table 3:** List of equipment used for the cardiovascular assessments.

Parameter	Equipment Used for Measurement	
cIMT	RS80 EVO device (Samsung Medison Co., Ltd., Seoul, Republic of Korea)	LA4-18B
cfIMT		LA3-12A
sfIMT		
ABI	Dopplex Ability Automatic ABI System (Huntleigh Healthcare Ltd., Cardiff, UK)	
TBI	Dopplex DMX Digital Doppler (Huntleigh Healthcare Ltd., Cardiff, UK)	
PWA parameters	Sphygmocor XCEL device (AtCorMedical, Sydney, Australia)	
cfPWV		

Abbreviations: cIMT—intima–media thickness (common carotid artery); cfIMT—intima–media thickness (common femoral artery); sfIMT—intima–media thickness (superficial femoral artery); ABI—ankle–brachial index; TBI—toe–brachial index; PWA—pulse wave analysis; cfPWV—carotid–femoral pulse wave velocity.

**Table 4:** Characteristics of the study population according to subgroup analysis (patients with and without metabolic syndrome).

Parameter	Without MetS (*N* = 55)	With MetS (*N* = 45)	*p*-Value
Age [years]	55.41 ± 19.54	62.94 ± 11.63	0.025
Women, *n* (%)	39 (70.9)	24 (53.3)	0.07
Men, *n* (%)	16 (29.1)	21 (46.7)	
BMI [kg/m^2^]	24.73 ± 4.28	29.80 ± 4.43	<0.001
Waist circumference [cm]	86.52 ± 13.76	102.33 ± 11.66	<0.001
WHR	0.88 ± 0.09	0.95 ± 0.09	<0.001
WHtR	0.52 ± 0.091	0.61 ± 0.064	<0.001
Body fat mass [kg]	19.48 ± 7.40	27.36 ± 8.07	<0.001
Body fat [%]	27.97 ± 7.95	32.17 ± 6.39	0.005
**Comorbidities**			
Overweight, *n* (%)	18 (32.7)	17 (37.8)	0.598
Obesity, *n* (%)	7 (12.7)	22 (48.9)	<0.001
Hypertension, *n* (%)	24 (43.6)	39 (86.7)	<0.001
Prediabetes, *n* (%)	5 (9.1)	16 (35.6)	0.0012
Diabetes, *n* (%)	2 (3.6)	20 (44.4)	<0.001
Chronic kidney disease, *n* (%)	0 (0.0)	2 (4.44)	Not applicable
Atrial fibrillation, *n* (%)	2 (3.6)	3 (6.7)	0.489
**Smoking status, *n*/*N* (%)**			
Current smoker	23/54 (42.6)	20/45 (44.4)	0.535
Former smoker	17/54 (31.5)	10/45 (22.2)	
Never-smoker	14/54 (25.9)	15/45 (33.3)	
**Laboratory measurements**			
Triglycerides [mg/dL]	91.0 (64.0; 123.0)	164.0 (120.0; 206.0)	<0.001
Non-HDL cholesterol [mg/dL]	126.6 (99.0; 147.0)	139.7 (118.0; 170.0)	0.046
Uric acid [mg/dL]	4.7 (3.3; 5.9)	5.6 (4.8; 7.1)	<0.001
HbA1c [%]	5.46 (5.29; 5.71)	5.88 (5.57; 6.14)	<0.001

Data are presented as mean ± standard deviation, median (Q1; Q3), or number (percentage), as appropriate. *p*-values refer to comparisons between participants with and without MetS. Smoking-status data were available for 99 participants. Abbreviations: BMI—body mass index; HbA1c—glycated hemoglobin; MetS—metabolic syndrome; WHR—waist-to-hip ratio; WHtR—waist-to-height ratio.

**Table 5:** The values of cardiovascular parameters in the study population.

Parameter	Mean ± SD/Median (Q1; Q3)	Minimum Value	Maximum Value
cIMT [mm]	0.828 (0.65; 1.0)	0.45	1.285
cfIMT [mm]	0.89 (0.618; 1.443)	0.38	5.4
sfIMT [mm]	0.6 (0.5; 0.785)	0.3	3.4
ABI	1.14 (1.053; 1.198)	0.615	1.285
TBI	0.8 (0.17)	0.33	1.17
cfPWV [m/s]	7.8 (6.7; 9.8)	4.0	23.2
cPP [mmHg]	38.0 (31.0; 49.0)	19.0	90.0
AP [mmHg]	10.0 (6.0; 18.0)	−3.0	52.0
AIx	26.65 (15.07)	−16.0	67.0
AIx75	23.6 (14.65)	16.0	67.0
HRD [ms]	875.0 (785.0; 1016.0)	539.0	1406.0
ED [ms]	301.84 (30.47)	245.0	373.0
SEVR [%]	160.32 (31.54)	86.0	246.0
ESP [mmHg]	110.0 (100.5; 120.0)	87.0	161.0

Abbreviations: cIMT—intima–media thickness (common carotid artery); cfIMT—intima–media thickness (common femoral artery); sfIMT—intima–media thickness (superficial femoral artery); ABI—ankle–brachial index; TBI—toe–brachial index; cfPWV—carotid–femoral pulse wave velocity; cPP—central pulse pressure; AP—augmentation pressure; AIx—augmentation index; AIx75—augmentation index normalized for heart rate of 75 per minute; HRD—heart rate duration; ED—ejection duration; SEVR—subendocardial viability ratio; ESP—end-systolic pressure.

**Table 6:** Correlations between cardiovascular parameter values and the number of metabolic syndrome (MetS) components.

Parameter	*N*	R	*p*
cIMT	98	0.371	<0.001
cfIMT	91	0.343	<0.001
sfIMT	92	0.516	<0.001
ABI	100	−0.072	0.476
TBI	100	0.171	0.09
cfPWV	97	0.398	<0.001
cPP	100	0.223	0.026
AP	100	0.041	0.687
AIx	100	−0.083	0.412
AIx75	99	−0.127	0.21
HRD [ms]	100	0.111	0.272
ED [ms]	100	−0.032	0.749
SEVR [%]	100	0.026	0.794
ESP [mmHg]	100	0.241	0.016

Abbreviations: cIMT—intima–media thickness (common carotid artery); cfIMT—intima–media thickness (common femoral artery); sfIMT—intima–media thickness (superficial femoral artery); ABI—ankle–brachial index; TBI—toe–brachial index; cfPWV—pulse wave velocity; cPP—central pulse pressure; AP—augmentation pressure; AIx—augmentation index; AIx75—augmentation index normalized for heart rate of 75 per minute; HRD—heart rate duration; ED—ejection duration; SEVR—subendocardial viability ratio; ESP—end-systolic pressure.

**Table 7:** Differences between patients with and without detectable atherosclerotic plaque.

Parameter	Patients with Atherosclerotic Plaque	Patients Without Atherosclerotic Plaque	*p*
	Mean ± SD/Median (Q1; Q3)	Mean ± SD/Median (Q1; Q3)	
Number of MetS components	3.0 (2.0; 4.0)	1.0 (0.0; 2.0)	<0.001 *
cIMT [mm]	0.95 (0.82; 1.065)	0.615 (0.565; 0.67)	<0.001 *
cfIMT [mm]	1.2 (0.85; 1.585)	0.585 (0.485; 0.65)	<0.001 *
sfIMT [mm]	0.678 (0.6; 0.85)	0.5 (0.4; 0.55)	<0.001 *
ABI	1.118 (1.04; 1.18)	1.168 (1.12; 1.215)	0.039 *
TBI	0.778 (0.181)	0.849 (0.152)	0.277 **
cfPWV [m/s]	9.0 (7.65; 10.5)	6.6 (5.8; 7.4)	<0.001 *
cPP [mmHg]	44.0 (35.0; 54.0)	31.0 (28.0; 37.0)	<0.001 *
AP [mmHg]	12.5 (8.0; 21.0)	6.5 (4.0; 10.0)	<0.001 *
AIx	29.77 (14.7)	20.59 (14.09)	0.003 **
AIx75	26.03 (14.52)	18.94 (13.97)	0.021 **
HRD [ms]	930.0 (797.0; 1039.0)	840.0 (776.0; 930.0)	0.081 *
ED [ms]	308.03 (33.09)	289.82 (20.06)	0.004 **
SEVR [%]	156.76 (31.85)	167.24 (30.19)	0.116 **
ESP [mmHg]	112.5 (103.0; 123.0)	105.0 (97.0; 113.0)	0.059 *

Abbreviations: MetS—metabolic syndrome; cIMT—intima–media thickness (common carotid artery); cfIMT—intima–media thickness (common femoral artery); sfIMT—intima–media thickness (superficial femoral artery); ABI—ankle–brachial index; TBI—toe–brachial index; cfPWV—pulse wave velocity; cPP—central pulse pressure; AP—augmentation pressure; AIx—augmentation index; AIx75—augmentation index normalized for heart rate of 75 per minute; HRD—heart rate duration; ED—ejection duration; SEVR—subendocardial viability ratio; ESP—end-systolic pressure; (*)—*p*-value according to U Mann–Whitney test; (**)—*p*-value according to Student’s *t*-test.

**Table 8:** Multivariate analysis model significance test for describing parameters related to subclinical atherosclerosis and arterial stiffness (AS). The model was adjusted for age, sex, and body mass index (BMI).

Parameter	Multiple R^2^	*p*
cIMT	0.601	<0.001
log(cfIMT)	0.487	<0.001
log(sfIMT)	0.476	<0.001
TBI	0.151	0.003
log(cfPWV)	0.556	<0.001
log(cPP)	0.508	<0.001
AIx	0.377	<0.001
AIx75	0.305	<0.001
HRD	0.117	0.018
ED	0.163	0.002
SEVR	0.119	0.016
ESP	0.164	0.002

Abbreviations: cIMT—intima–media thickness (common carotid artery); cfIMT—intima–media thickness (common femoral artery); sfIMT—intima–media thickness (superficial femoral artery); TBI—toe–brachial index; cfPWV—carotid–femoral pulse wave velocity; cPP—central pulse pressure; AP—augmentation pressure; AIx—augmentation index; AIx75—augmentation index normalized for heart rate of 75 per minute; HRD—heart rate duration; ED—ejection duration; SEVR—subendocardial viability ratio; ESP—end-systolic pressure.

**Table 9:** Multivariate analysis of the impact of the number of metabolic syndrome (MetS) components on parameters related to subclinical atherosclerosis and arterial stiffness (AS). The model was adjusted for age, sex, and body mass index (BMI).

Parameter	*N*	Standardized β	95% CI	Nominal *p*-Values	BH-Adjusted *p*-Value
cIMT	98	0.102	−0.062; 0.267	0.218	0.374
log(cfIMT)	91	0.188	−0.005; 0.381	0.056	0.236
log(sfIMT)	92	0.076	−0.118; 0.269	0.439	0.585
TBI	100	0.041	−0.201; 0.283	0.738	0.805
log(cfPWV)	97	−0.075	−0.255; 0.106	0.414	0.585
log(cPP)	100	−0.052	−0.236; 0.132	0.575	0.690
AIx	100	−0.148	−0.356; 0.059	0.159	0.374
AIx75	99	−0.212	−0.433; 0.008	0.059	0.236
HRD	100	0.167	−0.08; 0.414	0.181	0.374
ED	100	−0.152	−0.393; 0.088	0.211	0.374
SEVR	100	0.33	0.084; 0.577	0.009	0.108
ESP	100	−0.026	−0.266; 0.215	0.833	0.833

Abbreviations: cIMT—intima–media thickness (common carotid artery); cfIMT—intima–media thickness (common femoral artery); sfIMT—intima–media thickness (superficial femoral artery); TBI—toe–brachial index; cfPWV—carotid–femoral pulse wave velocity; cPP—central pulse pressure; AIx—augmentation index; AIx75—augmentation index normalized for heart rate of 75 per minute; HRD—heart rate duration; ED—ejection duration; SEVR—subendocardial viability ratio; ESP—end-systolic pressure. β represents the standardized regression coefficient for the number of MetS components.

**Table 10:** Results of multivariate analysis to examine the relationship between subendocardial viability ratio (SEVR) and the number of metabolic syndrome (MetS) components in different model designs.

Model of the Relationship Between SEVR and the Number of MetS Components	*N*	Multiple R^2^	*p* Omnibus	β	95% CI	*p*
Unadjusted	100	0.005	0.48	0.071	−0.128; 0.271	0.48
Adjusted for sex	100	0.005	0.765	0.068	−0.137; 0.272	0.514
Adjusted for age	100	0.056	0.059	0.166	−0.046; 0.377	0.124
Adjusted for BMI	100	0.064	0.041	0.229	−0.003; 0.461	0.053
Adjusted for age and BMI	100	0.119	0.007	0.331	0.089; 0.572	0.008
Adjusted for sex, age, and BMI	100	0.119	0.016	0.33	0.084; 0.577	0.009

## Data Availability

The data presented in this study are available upon request from the corresponding author.
